# Supplementary material for: Dynamic Physiological Phenotyping of Drought-Stressed Pepper Plants Treated With “Productivity-Enhancing” and “Survivability-Enhancing” Biostimulants
Source: Front Plant Sci. 2019 Jul 17;10:905. doi: 10.3389/fpls.2019.00905 (PMC6654182; doi:10.3389/fpls.2019.00905)
Supplement: Supplementary file 2 [file Table_1.docx]

**Supplementary Table 1: Steel–Dwass test for Figure 3A**

| **Days** | ***p*-values** | | | | | |
| --- | --- | --- | --- | --- | --- | --- |
|  | **Drought** | | | **Well irrigated** | | |
|  | Control  With  ICL-SW | Control  With  ICL-NewFo1 | ICL-SW  With  ICL-NewFo1 | Control  With  ICL-SW | Control  With  ICL-NewFo1 | ICL-SW  With  ICL-NewFo1 |
| 1 – 7 | *p*>0.05 | *p*>0.05 | *p*>0.05 | *p*>0.05 | *p*>0.05 | *p*>0.05 |
| 8 | *p*>0.05 | *p*>0.05 | *p*<0.05 | *p*<0.05 | *p*>0.05 | *p*<0.05 |
| 9 | *p*>0.05 | *p*>0.05 | *p*>0.05 | *p*>0.05 | *p*>0.05 | *p*>0.05 |
| 10 | *p*>0.05 | *p*>0.05 | *p*>0.05 | *p*>0.05 | *p*>0.05 | *p*>0.05 |
| 11 | *p*>0.05 | *p*>0.05 | *p*>0.05 | *p*>0.05 | *p*>0.05 | *p*>0.05 |
| 12 | *p*>0.05 | *p*>0.05 | *p*>0.05 | *p*>0.05 | *p*>0.05 | *p*>0.05 |
| 13 | *p*>0.05 | *p*>0.05 | *p*>0.05 | *p*>0.05 | *p*>0.05 | *p*>0.05 |
| 14 | *p*>0.05 | *p*>0.05 | *p*>0.05 | *p*>0.05 | *p*>0.05 | *p*>0.05 |
| 15 | *p*>0.05 | *p*>0.05 | *p*>0.05 | *p*>0.05 | *p*>0.05 | *p*>0.05 |
| 16 | *p*>0.05 | *p*>0.05 | *p*>0.05 | *p*>0.05 | *p*>0.05 | *p*>0.05 |
| 17 | *p*>0.05 | *p*>0.05 | *p*>0.05 | *p*>0.05 | *p*>0.05 | *p*>0.05 |
| 18 | *p*>0.05 | *p*>0.05 | *p*>0.05 | *p*<0.05 | *p*>0.05 | *p*>0.05 |
| 19 – 36 | *p*>0.05 | *p*>0.05 | *p*>0.05 | *p*>0.05 | *p*>0.05 | *p*>0.05 |

**Supplementary Table 2: Steel–Dwass test for Figure 3B**

| **Days** | ***p*-values** | | |
| --- | --- | --- | --- |
|  | **Drought** | | |
|  | Control  With  ICL-SW | Control  With  ICL-NewFo1 | ICL-SW  With  ICL-NewFo1 |
| 1 – 36 | *p*>0.05 | *p*>0.05 | *p*>0.05 |

**Supplementary Table 3: Steel–Dwass test for Figure 3D**

| **Time (h)** | ***p*-values** | | | | | |
| --- | --- | --- | --- | --- | --- | --- |
|  | **Drought** | | | **Well irrigated** | | |
|  | Control  With  ICL-SW | Control  With  ICL-NewFo1 | ICL-SW  With  ICL-NewFo1 | Control  With  ICL-SW | Control  With  ICL-NewFo1 | ICL-SW  With  ICL-NewFo1 |
| 0600 | *p*>0.05 | *p*>0.05 | *p*>0.05 | *p*<0.05 | *p*<0.05 | *p*>0.05 |
| 0700 | *p*<0.05 | *p*>0.05 | *p*>0.05 | *p*>0.05 | *p*>0.05 | *p*>0.05 |
| 0800 | *p*>0.05 | *p*>0.05 | *p*>0.05 | *p*<0.05 | *p*>0.05 | *p*>0.05 |
| 0900 | *p*>0.05 | *p*>0.05 | *p*<0.05 | *p*<0.05 | *p*>0.05 | *p*>0.05 |
| 1000 | *p*>0.05 | *p*>0.05 | *p*<0.05 | *p*<0.05 | *p*>0.05 | *p*>0.05 |
| 1100 | *p*>0.05 | *p*>0.05 | *p*>0.05 | *p*<0.05 | *p*>0.05 | *p*>0.05 |
| 1200 | *p*<0.05 | *p*>0.05 | *p*<0.05 | *p*<0.05 | *p*>0.05 | *p*>0.05 |
| 1300 | *p*<0.05 | *p*>0.05 | *p*<0.05 | *p*<0.05 | *p*>0.05 | *p*>0.05 |
| 1400 | *p*<0.05 | *p*>0.05 | *p*<0.05 | *p*<0.05 | *p*>0.05 | *p*>0.05 |
| 1500 | *p*<0.05 | *p*>0.05 | *p*<0.05 | *p*<0.05 | *p*>0.05 | *p*>0.05 |
| 1600 | *p*<0.05 | *p*>0.05 | *p*<0.05 | *p*<0.05 | *p*>0.05 | *p*>0.05 |
| 1700 | *p*>0.05 | *p*>0.05 | *p*<0.05 | *p*<0.05 | *p*>0.05 | *p*<0.05 |
| 1800 | *p*<0.05 | *p*>0.05 | *p*<0.05 | *p*<0.05 | *p*>0.05 | *p*<0.05 |
| 1900 | *p*>0.05 | *p*>0.05 | *p*>0.05 | *p*<0.05 | *p*>0.05 | *p*<0.05 |

**Supplementary Table 4: Steel–Dwass test for Figure 4A**

| **Days** | ***p*-values** | | | | | |
| --- | --- | --- | --- | --- | --- | --- |
|  | **Drought** | | | **Well irrigated** | | |
|  | Control  With  ICL-SW | Control  With  ICL-NewFo1 | ICL-SW  With  ICL-NewFo1 | Control  With  ICL-SW | Control  With  ICL-NewFo1 | ICL-SW  With  ICL-NewFo1 |
| 1 – 36 | *p*>0.05 | *p*>0.05 | *p*>0.05 | *p*>0.05 | *p*>0.05 | *p*>0.05 |

**Supplementary Table 5: White test for Figure 4C.**

**Supplementary Table 6: White test for Figure 6B**


**Supplementary Table 7: White test for Supplementary Figure 6.**

**Supplementary Table 8: White test for Supplementary Figure 7.**
